# Clinical and Physiological Correlates of Irritability in Depression: Results from the Netherlands Study of Depression and Anxiety

**DOI:** 10.1155/2011/126895

**Published:** 2011-10-26

**Authors:** Floor E. A. Verhoeven, Linda Booij, Nic J. A. Van der Wee, Brenda W. H. J. Penninx, A. J. Willem Van der Does

**Affiliations:** ^1^Faculty of Social and Behavioural Sciences, Institute of Psychology, Leiden University, Wassenaarseweg 52, 2333 AK Leiden, The Netherlands; ^2^Sainte-Justine Hospital Research Center, 3175 Chemin de la Côte Sainte-Catherine, Montreal, QC, Canada H3T 1C5; ^3^Department of Psychiatry, University of Montreal, 2900 Boulevard Édouard-Montpetit, Montreal, QC, Canada H3T 1J4; ^4^Department of Psychiatry, Leiden University Medical Center, Albinusdreef 2, 2333 ZA Leiden, The Netherlands; ^5^Department of Psychiatry, VU University Medical Center, A.J. Ernststraat 1187, 1081 HL Amsterdam, The Netherlands; ^6^Department of Psychiatry, University Medical Center Groningen, Hanzeplein 1, 9700 RB Groningen, The Netherlands

## Abstract

*Objective*. Irritable and nonirritable depressed patients differ on demographic and clinical characteristics. We investigated whether this extends to psychological and physiological measures. *Method*. We compared irritable and nonirritable unipolar depressed patients on symptomatology, personality, and (psycho)physiological measures (cortisol, cholesterol, and heart rate variability). Symptomatology was reassessed after one year, and we also compared depressed patients who were irritable or non-irritable at both time points (Irr++ versus Irr−−). *Results*. Almost half (46%; *N* = 420) of the sample was classified as irritable. These patients scored higher on depression severity, anxiety, hypomanic symptoms, and psychological variables. No differences were observed on physiological markers after correction for depression severity. The same pattern was found when comparing Irr++ and Irr−− groups. *Conclusion*. Irritable and non-irritable depressed patients differ on clinical and psychological variables, but not on the currently investigated physiological markers. The clinical relevance of the distinction and the significance of the hypomanic symptoms remain to be demonstrated.

## 1. Introduction

Not all symptoms that are prevalent in major depression are part of its diagnostic criteria [1]. For instance, most depressed patients experience significant levels of anxiety. Irritability is also reported by many. In children, irritability is the most common symptom of depression [2] and is one of the diagnostic criteria. Two recent studies have examined the clinical significance of irritability in depression in adults. In a community sample of depressed patients, approximately half of the 955 patients with lifetime unipolar depression were also irritable during their worst episode [3], as measured with one item of the Composite International Diagnostic Interview. Depressed patients scoring positive on irritability had a younger age of onset and higher rates of comorbid attention-deficit/hyperactivity disorder, oppositional-defiant disorder, intermittent explosive disorder, dysthymia and anxiety disorders [3]. A second study [4] compared depressed patients with and without irritability based on the irritability item of a standardized symptom interview. Irritable depressed patients (*N* = 1, 067; 46%) reported more anxiety, loneliness and annoyance by daily hassles, and more prior suicide attempts than nonirritable depressed patients. 

Previous research by the same group focused on a depression subtype characterized by the presence of anger attacks, in other words by disturbances in the regulation of irritability [5–7]. Around 40% of depressed outpatients had one or more anger attacks during the past month [8, 9]. In comparison with depressed patients without anger attacks, these patients had higher levels of hostility, anxiety, somatization [9], higher cholesterol levels [10], more axis II psychopathology [11], increased risk of cardiac dysfunction [12], and a younger age of depression onset [13]. Depressed patients with anger attacks also showed a blunted response to the serotonin (5-HT) agonist fenfluramine [14]. Compared to healthy controls, depressed patients with anger attacks showed differential activation of orbitofrontal–limbic circuits following an anger induction task [15].

It has been suggested that irritability may also be a feature of unrecognized bipolar (spectrum) disorder [16–18]. It has not been investigated yet to what extent irritability during a depressive episode predicts the development of bipolar disorder. However, other features of bipolar disorder, such as early age of onset, suicidality, family history, greater episode recurrence, and atypical depression were not found to be more common in the irritable depressed [4]. 

The present study had two aims. The first aim was to investigate whether the psychological and biological profile that has been found for depressed patients with anger attacks also applies to depressed patients with irritability. We compared outpatients with irritable and nonirritable unipolar depression on demographic, clinical, psychological, and biological markers that had previously been associated with impulsivity, aggression, or anger attacks. These markers include personality, cognitive reactivity, heart rate variability [12], cholesterol [10], and cortisol [19]. We hypothesized that depressed patients reporting irritability would score higher on anxiety and suicidality, lower on agreeableness, and higher on aggression reactivity. We also expected that irritable depression would be associated with lower heart rate variability, lower cholesterol concentrations, and higher levels of cortisol, particularly the cortisol awakening rise. The second aim was to investigate the association of irritability in depression with features of (hypo-)mania. For this, we measured (hypo-)manic symptoms in the same patients.

## 2. Methods

### 2.1. Participants

Participants were selected from the Netherlands Study of Depression and Anxiety (NESDA). This cohort study follows 2,981 adult participants over the course of 8 years [20]. NESDA respondents were recruited from the community and through primary and secondary care facilities. The total NESDA sample contains 2,329 individuals with a lifetime diagnosis of depression, dysthymia and/or an anxiety disorder, and 652 healthy participants. Participants with a diagnosis of bipolar disorder, a psychotic disorder, obsessive compulsive disorder, or severe addiction disorder are excluded from NESDA. For the present study, we selected the 913 participants who met the criteria for major depression, minor depression, or dysthymia during the month prior to study admission.

### 2.2. Instruments

#### 2.2.1. Diagnoses

Current and past DSM-IV diagnoses of mood disorders, anxiety disorders, and alcohol abuse and dependency were assessed with the Composite International Diagnostic Interview (CIDI) [21]. The interviews were conducted and scored by trained and supervised clinical research staff. Psychotic disorders and addictions were assessed in an open interview and checked in the medical records. Severity of alcohol dependence and abuse was assessed with the Audit questionnaire [22].

#### 2.2.2. Symptomatology

Severity of depression during the past week was defined as the total score on the Inventory of Depressive Symptomatology (IDS-SR) [23], excluding the irritability item. Anxiety symptoms were assessed with the Beck Anxiety Index (BAI) [24]. The BAI measures the somatic and cognitive aspects of anxiety during the past week (e.g., “numbness or tingling” and “fear of the worst happening”). It contains 21 items, scored on a four-point scale. The symptoms of bipolar disorder were assessed with the Mood Disorder Questionnaire (MDQ) [25], which contains 13 items derived from DSM-IV criteria and clinical experience (e.g., “you were so irritable that you shouted at people or started fights or arguments” and “you felt more self-confident than usual”).

#### 2.2.3. Irritability

Irritability status was determined using one item from the Inventory of Depressive Symptomatology—Self-Report (IDS-SR) [23]. This item asks whether the participant has been “feeling irritable during the past seven days”. The answers are scored on a four-point scale with descriptors “not feeling irritable” (1), “feeling irritable less than half the time” (2), “feeling irritable more than half the time” (3), or “feeling extremely irritable nearly all of the time” (4). The sample was split into low (scoring 1 or 2) (Irr−) and high irritable groups (scoring 3 or 4) (Irr+). The validity of this criterion has been shown previously in other cohorts [3, 4], but since the IDS measures past week severity only, irritability was reassessed at a one-year followup. This allowed us to create somewhat more extreme subgroups of depressed patients who were irritable both at baseline and at one-year followup (Irr++) and patients who were nonirritable on both assessments (Irr−−).

#### 2.2.4. Suicidality

Previous suicide attempts were assessed with the Beck scale for suicide ideation [26]. 

#### 2.2.5. Psychological Variables

Cognitive vulnerability to depression was measured with the Leiden Index of Depression Sensitivity—Revised (LEIDS-R) [27, 28]. The LEIDS-R, a 34-item self-report scale, measures cognitive reactivity to sad mood on the following subscales: hopelessness/suicidality, acceptance/coping, aggression, control/perfectionism, risk aversion and rumination. Personality traits were assessed with the short form of the NEO Five Factor Inventory (NEO-FFI) [29]. 

### 2.3. Physiological Variables

#### 2.3.1. Cortisol Awakening Rise

Cortisol awakening response was used to investigate HPA-axis function [30]. During the baseline assessment, patients were instructed to collect four cortisol samples on a regular (working) day shortly after the interview. Samples were taken at awakening and at 30, 45, and 60 minutes after the first sample, after which they were returned by mail after collection. Median time between the interview and saliva sampling was 9.0 days (25th–27th percentile, 4–22 days). Outcome measures were the area under the curve with respect to the ground (AUCg) and the area under the curve with respect to the increase (AUCi) [31, 32]. 

#### 2.3.2. Cholesterol

Total cholesterol, low-density lipoprotein (LDL) and high-density lipoprotein (HDL), and cholesterol levels (both measures of serum cholesterol) were assayed from a blood sample taken after an overnight fast [33].

#### 2.3.3. Heart Rate Variability (HRV)

Heart rate variability (HRV) was assessed using a VU-AMS monitoring system [34] which was worn during most of the baseline measurement (average registration 99.9 minutes). The various phases of the session (resting baseline, interviews, and a cognitive task) were marked using an event button. Mean heart rate, standard deviation of the interbeat intervals (SDNN), and the different measures of respiratory sinus arrhythmia (RSA) were calculated from the interbeat interval (IBI) time series and respiration signal [34–36]. For the current study, we investigated SDNN and RSA measured in (supine) rest condition (during which no interview was conducted) and during performance of a cognitive test (test condition) [37].

### 2.4. Data Screening

Data were checked for missings and outliers, normality of distributions, and homogeneity of variances. 145 of the 913 participants did not return their questionnaire package resulting in 145 missings on all variables of the LEIDS-R. On the age of onset variable, 162 participants had missing values. In about half of the other variables, a low number of missing values (<20) were replaced with the series mean of their subgroup (Irr+/Irr−) according to Tabachnik and Fidell [38]. The dataset was complete for the other variables. After removal of outliers (scores higher or lower than 2 standard deviations from the mean) [32], cortisol data were normally distributed. There were no statistical outliers (based on Cook's distances and studentized residuals) on any of the other variables.

### 2.5. Statistical Analyses

Group differences (Irr+ versus Irr−) were investigated by general linear models (GLMs). This was done in two steps. First, data were explored by using separate univariate GLMs with group (Irr+/Irr−) as the between subjects factor. Alpha was set at 0.05, however all outcome variables significant at the *P* < .15 level in these univariate analyses were entered in a multivariate GLM to take into account correlations among the dependent variables. Covariates were included in the univariate and multivariate analyses in order to reduce error variance [39]. The choice of covariates in each of the analyses was based on literature review and results of previous studies conducted in NESDA [37, 40]. Age, gender, and current depression symptoms were included as covariates in the GLMs for the LEIDS-R and NEO-FFI subscale and total scores. For the cortisol measures, we entered physical activity, smoking, cardiovascular disease, whether the participant was working on the day of data collection and hours of daylight in the month of data collection as covariates. In the HRV and cholesterol analyses, covariates were age, gender, depression severity, alcohol dependence and abuse, use of antidepressants, and heart medication. Participants were classified as nonsmoker, former smoker, smoker, or heavy smoker (>20 tobacco consumptions a day), and similar categories were made for alcohol use: nondrinker, mild drinker (<7 units/week), moderate drinker (7–14 u/wk), and heavy drinker (≥15 u/wk). Energy spent on physical activity per week was measured with the International Physical Activity Questionnaire [41]. Chi-square statistics were used in case of categorical variables. Logistic regression analysis was used to control for potential confounders in relationships involving categorical variables.

## 3. Results

### 3.1. Demographic and Clinical Characteristics

Univariate analyses showed that the irritable depressed group was significantly older than the nonirritable depressed group (*F*(1,911) = 10.7; *P* = .001). There was no significant difference in the distribution of males and females over the two groups (*χ* ^2^(1) = .004; *P* = .95). Participants in the Irr+ group had been recruited from specialized mental health institutions more often than participants in the Irr− group (*χ* ^2^(2) = 16.4; *P* < .001). 

Table 1 shows that the Irr+ group also had notably higher scores on severity of depression (IDS total minus Item 6; Irritability) (*F*(1,911) = 232.9; *P* < .001) than the Irr− group. This pattern was present at each recruitment site, with 9 points difference between Irr+ and Irr− in primary care (*F*  (1,367) = 76.6; *P* < .001) and specialized mental health care (*F*(1,466) = 102.3; *P* < .001) and 13 points difference in the general population (*F*  (1,72) = 47.5; *P* < .001). Irr+ participants also had higher anxiety (BAI total) symptoms (*F*(1,911) = 134.2; *P* < .001) and more lifetime anxiety disorders (*χ* ^2^(1) = 5.5; *P* = .019). Current GAD (*χ* ^2^(1) = 14.7; *P* < .001), panic disorder (*χ* ^2^(1) = 22.8; *P* < .001), and social anxiety disorder (*χ* ^2^(1) = 17.3; *P* < .001) were also more prevalent in the Irr+ group.

More patients in the Irr+ group had previously attempted suicide than patients in the Irr− group (25% versus 17%; *χ* ^2^(1) = 8.3; *P* = .004). However, an additional logistic regression analysis showed that the association between irritability and suicidality was no longer statistically significant after controlling for depression severity. Patients in the Irr+ group scored higher on three mania items of the MDQ: talkativeness (*χ* ^2^(1) = 4.03; *P* = .045), racing thoughts (*χ* ^2^(1) = 10.47; *P* = .001), and distractibility (*χ* ^2^(1) = 14.57; *P* < .001). 

### 3.2. Cognitive Vulnerability

The Irr+ group scored higher on all subscales of the LEIDS-R, with exception of the acceptance subscale. The Irr+ group also had a significantly higher LEIDS-R total score (*F*(1,766) = 68.8; *P* < .001). After adding age, gender, and IDS total score as covariates, only the difference between the scores on the aggression subscale of the LEIDS-R remained significant (*F*(1,763) = 39.4; *P* < .001).

### 3.3. Personality

The Irr+ group had significantly higher neuroticism scores (*F*(1,908) = 116.06; *P* < .001) and scored significantly lower on extraversion (*F*(1,908) = 39.48; *P* < .001), openness (*F*(1,909) = 6.71; *P* = .010), agreeableness (*F*(1,908) = 48.58; *P* < .001), and conscientiousness (*F*(1,908) = 5.38; *P* = .021) than the Irr- group. After correcting for age, gender, and total IDS score, the differences on neuroticism and agreeableness remained statistically significant.

### 3.4. Multivariate Analyses

The multivariate analyses (shown in Table 2) yielded similar results, with significant differences between Irr+ and Irr− on BAI total score (*F*(1,619) = 4.84; *P* = .028), LEIDS-R aggression (*F*(1,619) = 48.40; *P* < .001), and agreeableness (*F*(1,619) = 14.96; *P* < .001). Neuroticism was significant at trend level (*F*(1,619) = 3.71; *P* = .055). 

#### 3.4.1. Physiological Variables

Table 3 shows the outcomes on the (psycho)physiological markers. There were no significant differences in cortisol awakening response (CAR) between the Irr− and Irr+ irritable group. HDL cholesterol was significantly higher in the Irr− group (*F*(1,911) = 5.69; *P* = .017), but this difference was no longer significant after entering the covariates gender, age, smoking, alcohol abuse and dependence, and antidepressant and heart-medication use. Both groups did not differ significantly on measures of HRV either (all *P*  values > .12), with and without correction for presence of cardiac disease or use of heart medication. Presence of cardiac disease (5.7% versus 6.7%) and use of heart medication (13.3% versus 16.2%) did not differ significantly between depressed patients with and without irritability.

#### 3.4.2. Irritability at One-Year Followup

Depressed patients who were irritable both at baseline and at one-year followup (Irr++) (*N* = 138) differed from depressed patients who were nonirritable on both baseline and one-year followup (Irr−−) (*N* = 202) on largely the same outcomes. Fewer patients in the Irr++ group were recruited from primary health care or the community (*χ* ^2^(1) = 5.67; *P* = .020), and they scored higher on comorbid anxiety disorders. Their depression severity (IDS minus irritability) was also higher (*F*(1,338) = 77.74; *P* < .001). They scored higher on aggression reactivity (*F*(1,319) = 36.35; *P* < .001) and total LEIDS-R score (*F*(1,319) = 3.94; *P* = .048), after correction for depression severity, age, and gender. Irr++ participants also had higher neuroticism (*F*(1,335) = 12.94; *P* < .001) and lower agreeableness (*F*(1,335) = 8.62; *P* = .004) scores, after correction for age, gender, and depression severity. No physiological differences were found between the Irr++ and Irr−− groups.

## 4. Discussion

The present study showed that approximately half of the patients with a primary diagnosis of unipolar depression also have high levels of irritability. This is consistent with earlier research [3, 4]. Other studies have shown that the prevalence of anger attacks in patients with unipolar depression is only slightly lower at approximately 40% [6, 9, 42]. These studies, however, concerned patients recruited from secondary care facilities. In the current study, almost 60% of the patients recruited from psychiatric outpatient departments were classified as irritable. Irritability has been defined as “a feeling characterized by reduced control of temper” which often results in verbal or behavioral aggression [43]. Although irritability should be distinguished from more violent forms of aggressive and assaultive behavior, milder and more severe forms of irritability (e.g., anger attacks) may lie on a continuum [43]. Future research may investigate the exact relationship between irritability during depression and its outward manifestations such as anger attacks.

### 4.1. Clinical Characteristics of Irritable versus Nonirritable Depression

Irritable depressed patients were more severely depressed than nonirritable depressed patients. The difference in IDS scores was 10 points, which is more than one standard deviation. The severity of anxiety symptoms and suicidality was also higher. Moreover, irritable depressed patients were more often diagnosed with a comorbid anxiety disorder. The onset of depression was approximately two years earlier in the irritable depressed. They were also somewhat older at study entry and were more often recruited from secondary care facilities. With regards to their psychological profile, differences between irritable and nonirritable depressed patients were observed on a broad range of personality traits and cognitive vulnerability indices. However, after correction for depression severity, irritable depressed patients only had higher scores of aggression reactivity and lower scores of the personality trait agreeableness. Although participants are categorized into high- and low-irritable groups on the basis of one symptom, the psychological profile observed in the present study supports the validity of the subgroups.

### 4.2. Physiological Differences between Irritable and Nonirritable Depression

We found no differences between irritable and nonirritable depressed patients on any of the physiological markers that were investigated. Although HDL cholesterol was significantly higher in nonirritable patients, this result was no longer significant after correction for several covariates. No differences were observed on measures of heart rate variability (HRV) and cortisol awakening rise (CAR). We subsequently investigated the possibility that these physiological markers are related to more stringently defined subtypes, by selecting participants who were also depressed at one-year followup and showed either high or low scores of irritability at both time points. This comparison produced exactly the same pattern of findings. Irritable depressed patients had a greater prevalence of anxiety disorders and higher depression severity and aggression reactivity. Again, no differences were found on any of the physiological measures after correction for overall depression severity. The absence of differences at the physiological level was unexpected since studies in healthy samples have found greater HPA-axis reactivity [44, 45] and increased cardiac reactivity as a function of hostility and aggression [46, 47]. However, in a population-based sample anxiety and hostility were not related with HRV but with baroreflex sensitivity, which may be a more sensitive measure of vagal activity [47]. In depressed patients, the studies that investigated cardiac and HPA-axis reactivity concern comparisons between patients with and without anger attacks. These studies found higher cholesterol concentrations in depressed patients with anger attacks [48]. We found no support for this finding in the present sample of irritable versus nonirritable depressed patients. 

In the present study, HRV was assessed at rest and during a task that required cognitive effort. Cortisol samples were collected at home. It has been suggested that depressed patients with anxiety-aggression behaviors also have a greater sensitivity to stress [49]. It could be that irritable depressed patients only show greater HPA-axis and cardiac reactivity when exposed to a more significant stressor than performing a cognitive test. This could be further tested using the same measures as in the present study in combination with laboratory stress or anger induction paradigms. 

The clinical and psychological characteristics observed in the irritable depressed patients resemble a subtype of depression proposed by Van Praag [49–51]. He stated that aggression combined with anxiety is the primary feature of a subtype of depression which he called “stressor-precipitated, cortisol-induced, serotonin-related, anxiety/aggression-driven” (SeCA) depression [19]. This subtype may be further characterized by increased 5-HT disturbances, in which low mood is a secondary symptom [50] and with aggression disturbances as primary symptom. Unfortunately, markers of 5-HT function were not available in the present study. 

### 4.3. Symptoms of Bipolar Disorder in Irritable versus Nonirritable Depression

We also observed differences between irritable and nonirritable depressed patients on three symptoms of hypomania as measured by the MDQ: distractibility, talkativeness, and racing thoughts. Future longitudinal analyses of NESDA participants may show whether irritability during depression is a risk factor for the development of bipolar disorder.

### 4.4. Strengths and Limitations of the Current Study

The current study had several strengths, including a rather large sample size of currently depressed patients. Diagnoses were determined using a standardized interview. Patients were recruited from different sites and facilities, which increases the generalizability of the findings. The cross-sectional design is a limitation. This was partially remediated by the inclusion of a one-year follow up measurement of the clinical variables, which allowed us to investigate a more stable subgroup of irritable depressed patients. Although other studies used the same method [3, 4], the fact that the distinction between irritable and nonirritable depressed was based on just one symptom can be seen as a limitation. We interpret the selective differences on cognitive reactivity and personality as supporting the validity of the distinction.

### 4.5. Future Directions

Our findings indicate several clinical differences between depressed patients with and without irritability. High levels of irritability during depression are associated with more severe depression, higher levels of anxiety, and more comorbid anxiety disorders. Anxiety during depression is associated with poorer outcome, including higher risk of chronic depression, poorer treatment response [52], and increased suicide incidence [53]. Moreover, we found higher levels of aggression reactivity, hopelessness, and disagreeableness in irritable depressed patients. Both hopelessness and aggression reactivity have been associated with increased suicide risk [54]. Therefore, it is important to continue the investigation into possible underlying mechanisms of this form of depression. There is evidence that depressed patients with aggression dysregulation problems have more pronounced 5-HT alterations [49, 50, 55–57]. Moreover, there is some evidence that depression in combination with aggression (hostility, anger) is partly under genetic control [58].

## 5. Conclusion

In this cross-sectional assessment, approximately half of depressed patients were classified as irritable. These patients differ from low-irritable depressed patients on several other aspects of the clinical presentation, including depression severity and comorbid anxiety. Personality and cognitive vulnerability measures also differ between these groups. Future longitudinal studies in depressed patients are needed to investigate the consequences of high levels of irritability in terms of risk of bipolar disorder, course of disease and treatment response.

## Figures and Tables

**Table 1:** Demographic and clinical characteristics of low and high irritable depressed patients: univariate results.

	Low irritable (*N* = 493)	High irritable (*N* = 420)	
*Demographic features *			
Age (mean ± SD)	43.4 ± 12.3	40.7 ± 11.7	*η* ^2^ = .012*
Sex (% female)	65.9	65.7	OR = 0.99 (CI: 0.75–0.30)
Recruited from:			
Specialized mental health care (%)	45.2	58.6	OR = 1.71** (CI: 1.32–2.23)
Community + primary care (%)	54.8	41.4	

*Clinical features*			
Age of onset (mean ± SD)^‡^	28.6 ± 13.1	26.6 ± 12.2	*η* ^2^ = .006*
Smoking (% yes)	43.4	48.1	OR = 1.21 (CI: 0.93–1.57)
Alcohol (% recent abuse and/or dependence)	8.5	8.1	OR = 0.95 (CI: 0.59–1.52)
Comorbid anxiety (CIDI diagnosis%)	73.6	80.2	OR = 1.45* (CI: 1.06–1.99)
GAD (%)	24.9	36.7	OR = 1.74** (CI: 1.31–2.31)
Panic disorder (%)	22.7	37.1	OR = 2.01** (CI: 1.51–2.68)
Social phobia (%)	26.2	39.0	OR = 1.82** (CI: 1.37–2.39)
IDS total score (mean ± SD)^a^	27.8 ± 10.1	37.7 ± 9.5	*η* ^2^ = .204**
First degree family history with depression (%)	84.4	85.0	OR = 1.05 (CI: 0.73–1.51)
Beck Anxiety Inventory (mean ± SD)	15.8 ± 9.4	23.8 ± 11.4	*η* ^2^ = .128**
Suicidality (% ≥1 attempt during lifetime)	17.4	25.2	OR = 1.60* (CI: 1.16–2.20)

	Low irritable (*N* = 202)	High irritable (*N* = 138)	

MDQ (% yes)			
Elated mood	28.8	32.0	OR = 1.20 (CI: 0.88–1.64)
Increased self-confidence	39.4	39.9	OR = 1.02 (CI: 0.76–1.37)
Less sleep needed	42.0	44.0	OR = 1.09 (CI: 0.81–1.45)
More and/or faster speech	50.7	57.5	OR = 1.34 (CI: 1.01–1.79)*
Racing thoughts	75.6	84.5	OR = 1.84 (CI: 1.27–2.68)*
Concentration problems	74.6	85.3	OR = 2.06 (CI: 1.41–3.00)**
More energy	44.6	45.7	OR = 1.06 (CI: 0.80–1.42)
Increased activity	49.8	48.7	OR = 0.98 (CI: 0.73–1.30)
Heightened sociability	23.9	28.4	OR = 1.27 (CI: 0.92–1.76)
Increased libido	29.1	32.8	OR = 1.20 (CI: 0.88–1.64)
Risk taking	25.8	32.8	OR = 1.41 (CI: 1.03–1.93)
Financial risk taking	13.4	18.2	OR = 1.45 (CI: 0.98–2.14)
^ b^NEO FFI (mean ± SD)			
Neuroticism	40.6 ± 6.6	45.2 ± 6.1	*η* ^2^ = .009*
Extraversion	33.9 ± 6.7	31.1 ± 6.6	*η* ^2^ = .001
Openness	31.1 ± 5.0	30.1 ± 6.0	*η* ^2^ = .003
Agreeableness	43.5 ± 5.1	41.0 ± 5.6	*η* ^2^ = .022**
Conscientiousness	35.3 ± 6.2	34.3 ± 6.3	*η* ^2^ = .001

	Low irritable (*N* = 427)	High irritable (*N* = 341)	

^ b^LEIDS-R (mean ± SD)			
Hopelessness	6.8 ± 4.8	9.2 ± 5.2	*η* ^2^ < .001
Acceptance	1.9 ± 2.2	2.0 ± 2.6	*η* ^2^ = .002
Aggression	5.0 ± 4.3	8.6 ± 5.1	*η* ^2^ = .049**
Control	6.4 ± 3.8	7.3 ± 4.1	*η* ^2^ < .001
Risk aversion	10.6 ± 4.5	12.0 ± 4.6	*η* ^2^ = .001
Rumination	11.4 ± 4.7	13.5 ± 4.2	*η* ^2^ = .002
Total score	42.2 ± 17.5	52.7 ± 17.3	*η* ^2^ = .004

*.05 < *P* >.001.

**P < .001.

^‡^ *N* = 751 (low irritable. *N* = 382, high irritable, *N* = 369).

^a^IDS-SR total score *minus* the score on the irritability item.

^b^Controlled for current symptoms (IDS-total minus item 6), gender, and age.

**Table 2:** Demographic and clinical characteristics of low and high irritable depressed patients—multivariate results^a^.

	Low irritable (*n* = 323)	High irritable (*n* = 301)	
Age of onset (mean ± SD)	29.0 ± 13.2	26.9 ± 12.4	*η* ^2^ = .001
Beck Anxiety Inventory (mean ± SD)	16.6 ± 9.5	23.9 ± 11.3	*η* ^2^ = .008**
LEIDS (mean ± SD)			
Aggression	5.0 ± 4.2	8.8 ± 5.1	*η* ^2^ = .073***
NEO FFI (mean ± SD)			
Neuroticism	41.2 ± 6.8	45.3 ± 6.1	*η* ^2^ = .006*
Openness	31.1 ± 5.1	30.6 ± 5.9	*η* ^2^ < .001
Agreeableness	44.0 ± 5.0	41.4 ± 5.6	*η* ^2^ = .024***

^∗^.085 < *P *>  .05.

**.05 <* P* >  .001.

***P <  .001.

^a^Controlled for current symptoms (IDS-total minus item 6), gender, and age.

**Table 3:** Psychophysiological measures.

	Low irritable (*n* = 294)	High irritable (*n* = 218 )	
Cortisol (mean ± SD)			
^a^AUCi	1.9 ± 4.9	2.1 ± 5.2	*η* ^2^ = .001
^b^AUCg	18.1 ± 6.2	18.2 ± 5.8	*η*2 < .001

	(*n* = 487 )	(*n* = 419)	

^ c^Cholesterol (mean ± SD)			
Total cholesterol	5.2 ± 1.1	5.1 ± 1.1	*η* ^2^ = .001
LDL cholesterol	3.2 ± 1.0	3.2 ± 1.0	*η* ^2^ < .001
HDL cholesterol	1.6 ± 0.4	1.6 ± 0.4	*η* ^2^ = .001

	(*n* = 470)	(*n* = 404)	

^ d^Heart rate variability (mean ± SD)			
RSA_rest_	41.9 ± 27.7	45.6 ± 29.6	*η* ^2^ = .002
RSA_test_	40.8 ± 23.0	43.4 ± 23.9	*η* ^2^ = .001
SDNN_ rest_	71.2 ± 30.9	73.3 ± 31.8	*η* ^2^ = .001
SDNN_test_	62.2 ± 23.1	63.9 ± 22.9	*η* ^2^ = .002

^a^Controlled for sex, physical activity, cardiovascular disease, time of awakening, and hours of sleep.

^b^Controlled for smoking, physical activity, cardiovascular disease, working on testing day, and hours of daylight in month of testing.

^c^Controlled for current symptoms (IDS-total minus item 6), sex, age, smoking, alcohol abuse and dependence, and antidepressant use.

^d^Controlled for current symptoms (IDS-total minus item 6), sex, age, smoking, alcohol abuse and dependence, antidepressant and heart medication-use, and heart disease.
